# Amniotic fluid embolism complicated by pulmonary embolism leading to multiple organ dysfunction: case report

**DOI:** 10.3389/fmed.2026.1795316

**Published:** 2026-04-01

**Authors:** Min Liu, Chaxiang Li, Haishuang Mei, Zhaohui Zhang, Guilan Ban, Tan Yayuan

**Affiliations:** 1 The First College of Clinical Medical Science, China Three Gorges University/ Yichang Central People’s Hospital, Yichang, Hubei, China; 2 College of Medicine and Health Sciences, China Three Gorges University, Yichang, Hubei, China

**Keywords:** acute respiratory failure, cardiogenic shock, multiple organ dysfunction, pulmonary embolism, pulmonary infection

## Abstract

**Rationale:**

Amniotic fluid embolism (AFE) is a rare and life-threatening obstetric emergency with an extremely high mortality rate. Pulmonary thromboembolism (PTE), a severe comorbidity of AFE in the puerperium, further exacerbates hemodynamic disturbance and multiple organ injury, posing great challenges to clinical diagnosis and targeted intervention. Relevant clinical diagnosis and treatment experience still need to be further supplemented and optimized.

**Patient concerns:**

A 37-year-old advanced-age parturient with a history of hysteroscopic adhesiolysis suffered a sudden onset of AFE following forceps-assisted delivery, complicated with PE and severe multiple organ dysfunction. The patient developed acute respiratory failure, cardiogenic shock, and coagulopathy in a short period with critical clinical manifestations, requiring emergent intensive care intervention.

**Diagnosis:**

Amniotic fluid embolism complicated with pulmonary embolism and multiple organ dysfunction syndrome (MODS).

**Interventions:**

A multidisciplinary team (MDT) formulated and implemented a comprehensive and individualized treatment regimen for the patient: invasive mechanical ventilation was administered for respiratory support; precise volume management guided by pulse indicator continuous cardiac output (PICCO) was adopted, combined with vasoactive agents and inotropic drugs for circulatory support; tranexamic acid (TXA) and component blood transfusion were given to correct coagulopathy, and risk-stratified anticoagulant therapy was promptly initiated after the recovery of coagulation function; standardized anti-infection therapy and preventive measures for thrombotic complications were implemented throughout the entire treatment course.

**Outcomes:**

After 72 h of intensive care and dynamic treatment adjustment, the patient’s hemodynamic and respiratory status improved significantly and stabilized, and she was successfully weaned from mechanical ventilation. At the 30-day follow-up after discharge, the patient achieved complete recovery of organ function with no long-term sequelae or related complications.

**Lessons learned:**

This case indicates that clinicians should maintain a high degree of vigilance for the possibility of complicated PE in AFE patients, especially those with high-risk factors such as advanced age and operative delivery. Early identification via standardized diagnostic tools, seamless collaboration of the MDT, precise organ function support based on real-time monitoring, and dynamic adjustment of the treatment regimen according to the patient’s condition are the core keys to improving the success rate of treatment and the prognosis of patients with AFE complicated with PTE.

## 1 Introduction

AFE refers to a severe syndrome endangering maternal and fetal safety, which occurs when amniotic fluid or fetal solid components enter the maternal bloodstream during childbirth, causing pulmonary embolism, shock, disseminated intravascular coagulation (DIC), renal failure, and other complications within a very short period (1). It is also a rare pregnancy and childbirth complication with extremely high mortality (2). Multiple pregnancies are also recognized as an established risk factor for AFE (3). Elderly parturients and operative delivery (especially instrumental delivery orceps-assisted delivery) have been identified as potential risk factors for AFE (4). Mechanical damage to the fetal membranes and maternal vascular bed during such procedures may facilitate the entry of amniotic fluid components into the maternal circulation; however, the pathophysiological mechanism underlying this phenomenon has not been fully elucidated (5). Clinically, AFE often presents with nonspecific symptoms that overlap with those of preeclampsia, pulmonary thromboembolism, or cardiogenic shock, leading to delayed diagnosis and subsequent exacerbation of adverse outcomes (6). Although the pathogenesis of AFE has not been fully clarified and is difficult to predict and prevent, early identification and rapid establishment of a response team remain key factors in improving the success rate of AFE treatment (7).

PTE constitutes the most prevalent critical comorbidity of AFE in the puerperium, with its incidence reaching approximately 0.45/1000 in AFE patients during the postpartum period (8). Clinically, it is classified into massive, submassive, and low-risk PTE based on hemodynamic stability and the presence or absence of right ventricular dysfunction (9). For its clinical diagnosis, CTA of the pulmonary artery is the gold standard confirmatory test, with preliminary screening relying on standardized and pregnancy-adapted assessment tools (10). The Wells scale is a classic clinical probability scoring system for preliminary screening of pulmonary embolism, which quantifies the risk of pulmonary embolism through 6 core indicators such as clinical symptoms, obstetric risk factors and alternative diagnoses (11). The patient in this case scored 6 points, according to the scale, which provided a quantitative basis for early suspicion of pulmonary embolism. The pregnancy-adapted YEARS criteria optimizes the diagnostic process for suspected pulmonary embolism in postpartum women, with three core clinical questions to guide the selection of subsequent imaging examinations (12), which enhances diagnostic efficiency while effectively mitigating the risk of external transfer for critically ill patients (13). In this case, since pulmonary embolism was identified as the most likely diagnosis based on clinical manifestations, CTA was directly performed according to the YEARS criteria, and the embolism in the left pulmonary artery branches was confirmed, realizing the early and accurate diagnosis of AFE complicated with pulmonary embolism. Both tools are widely used in the clinical differential diagnosis of AFE complicated by pulmonary embolism, providing a standardized and evidence-based basis for early identification and targeted intervention.

## 2 Case presentation

A 37-year-old female was admitted for antenatal care in March 2024. She had a history of 3 pregnancies and 0 live births, with a body mass index (BMI) of 20.03 kg/m^2^, and no smoking, drinking, drug abuse, or allergy history. In 2021, she underwent hysteroscopic uterine adhesiolysis in this hospital. No abnormalities were found in the prenatal examinations during this pregnancy. On the 2nd day after admission, at 18:19, a live infant was delivered in cephalic presentation via forceps-assisted delivery under epidural anesthesia, with intact placenta and fetal membranes. After delivery, 10 U of oxytocin were administered intravenously to promote uterine contraction; at 19:41, the patient developed restlessness, shortness of breath, cough, weakness, cyanosis of the lips, and a sense of impending death, with no epileptic seizures noted. Her pulse oxygen saturation (SpO₂) dropped to 85%, heart rate was 120–130 beats/min, blood pressure was 66/33 mmHg (1 mmHg = 0.133 kPa), and respiratory rate was 30–40 breaths/min; the uterine fundus was at the level of the umbilicus, with minimal vaginal bleeding (approximately 300 mL) and a small amount of blood clots. AFE was suspected, and the patient was transferred to the intensive care unit (ICU) after bedside emergency treatment.

On physical examination upon ICU admission, the patient was in a sedated and analgesic state. The Richmond Agitation-Sedation Scale (RASS) score was −4, and the Critical-Care Pain Observation Tool (CPOT) score was 0. The diameter of both pupils was 2/2 mm, with positive light reflex; body temperature was 36.6 °C, heart rate was 120 beats/min, she was on mechanical ventilation (FiO₂ 100%), blood pressure was 101/54 mmHg (maintained by intravenous infusion of norepinephrine at 2 μg/(kg·min) and metaraminol at 5 μg/(kg·min)), and SpO₂ was 92%. Auscultation revealed coarse breath sounds and obvious rales in both lungs, regular heart rhythm, and no murmurs heard at all cardiac valve auscultation areas; the abdomen was flat and soft, bowel sounds were weak, and the patient was uncooperative with tenderness and rebound tenderness examination. The liver and spleen were not palpable below the costal margin; there was no edema in either lower limb, physiological reflexes were present, and pathological reflexes were absent. The patient presented with cardiogenic shock, respiratory failure requiring mechanical ventilation, and abnormal hematological parameters, with clinical manifestations highly suggestive of AFE complicated by PTE. To quantify the clinical probability of PTE and optimize the diagnostic workflow, the Wells scale and pregnancy-adapted YEARS criteria were sequentially used for diagnostic evaluation of the patient. Wells scale assessment: The patient was scored using the 6 core indicators of the Wells scale. She had positive PTE-related clinical symptoms (dyspnea, chest tightness), definite obstetric risk factors (puerperium), and no alternative diagnosis with a higher clinical probability than PTE, with a total score of 6 points (indicating moderate-to-high clinical probability of PTE). This quantitative result provided a clear basis for the clinical team to initially suspect PTE and decide on further imaging studies. YEARS criteria assessment: Based on the 3 core clinical questions in the pregnancy-adapted YEARS criteria, clinical evaluation confirmed PTE as the most likely diagnosis for the patient, with no signs of deep vein thrombosis ruled out and no low clinical probability of PTE. In addition, to assess the severity of the patient’s condition and the degree of multiple organ dysfunction, the Acute Physiology and Chronic Health Evaluation II(APACHE II) and Sequential Organ Failure Assessment (SOFA) scales were applied. Based on the worst acute physiological indicators within 24 h of admission, combined with age and chronic health status, the APACHEII score was 16 points: the acute physiological score was 16 points, derived from abnormal indicators including tachycardia, acidosis, decreased hematocrit, elevated white blood cell count, and decreased level of consciousness; the age score was 0 points; the chronic health score was 0 points, as the patient had no history of severe chronic diseases (e.g., severe liver failure, dialysis dependence, immunosuppression, severe cardiopulmonary diseases) and no daily nursing dependence. This score indicated a moderate-to-severe condition. The baseline SOFA score was 11 points, indicating severe multiple organ dysfunction involving the respiratory, cardiovascular, coagulation, hepatic, and nervous systems: respiratory system score, 1 point; cardiovascular system score, 4 points; coagulation system score, 1 point; hepatic system score, 1 point; nervous system score, 4 points; renal system score, 0 points (no obvious dysfunction detected on admission). Laboratory tests showed the following results: Routine blood test: white blood cell count 19.70 × 10^9^/L, red blood cell count 3.16 × 10^12^/L, hemoglobin 69 g/L, hematocrit 20.2%, platelet count 109 × 10^9^/L, neutrophil count 18.38 × 10^9^/L, and neutrophil percentage 93.3%. Prothrombin time (PT) 16.6 s, prothrombin activity (PTA) 63.0%, prothrombin international normalized ratio (INR) 1.33; Thrombin-antithrombin III complex (TAT) 22.51 ng/mL, plasmin-*α*₂ plasmin inhibitor complex (PIC) 6.04 μg/mL, tissue plasminogen activator/plasminogen activator inhibitor-1 complex (tPAIC) 36.729 ng/mL, Fibrinogen level (Fib)4.29 (g/L); D-dimer 28.8 μg/mL; 3P test (protamine paracoagulation test) was positive. Creatine kinase (CK) 418 IU/L, creatine kinase-MB (CK-MB) 58 U/L, cardiac troponin I (TnI) 0.032 μg/L, N-terminal pro-B-type natriuretic peptide (NT-proBNP) 486 ng/L. Total bilirubin (TBIL) 25.0 μmol/L, direct bilirubin (DBIL) 10.9 μmol/L, albumin (ALB) 29.22 g/L, calcium (Ca) 1.74 mmol/L. Creatinine 52 μmol/L, blood urea nitrogen 2.78 mmol/L. Carbon dioxide combining power 16.0 mmol/L, C-reactive protein (CRP) 8.19 mg/L, procalcitonin (PCT) 2.08 ng/mL, interleukin-6 (IL-6) 47.4 pg./mL. Arterial blood gas analysis: pH 7.26, arterial partial pressure of oxygen (PO₂) 325.57 mmHg, arterial partial pressure of carbon dioxide (PCO₂) 35 mmHg, lactic acid (Lac) 5.55 mmol/L, bicarbonate (HCO₃^−^) 13.6 mmol/L, base excess (BE) -12.24 mmol/L.

## 3 Treatment

### 3.1 Respiratory support treatment

**Figure 1:**
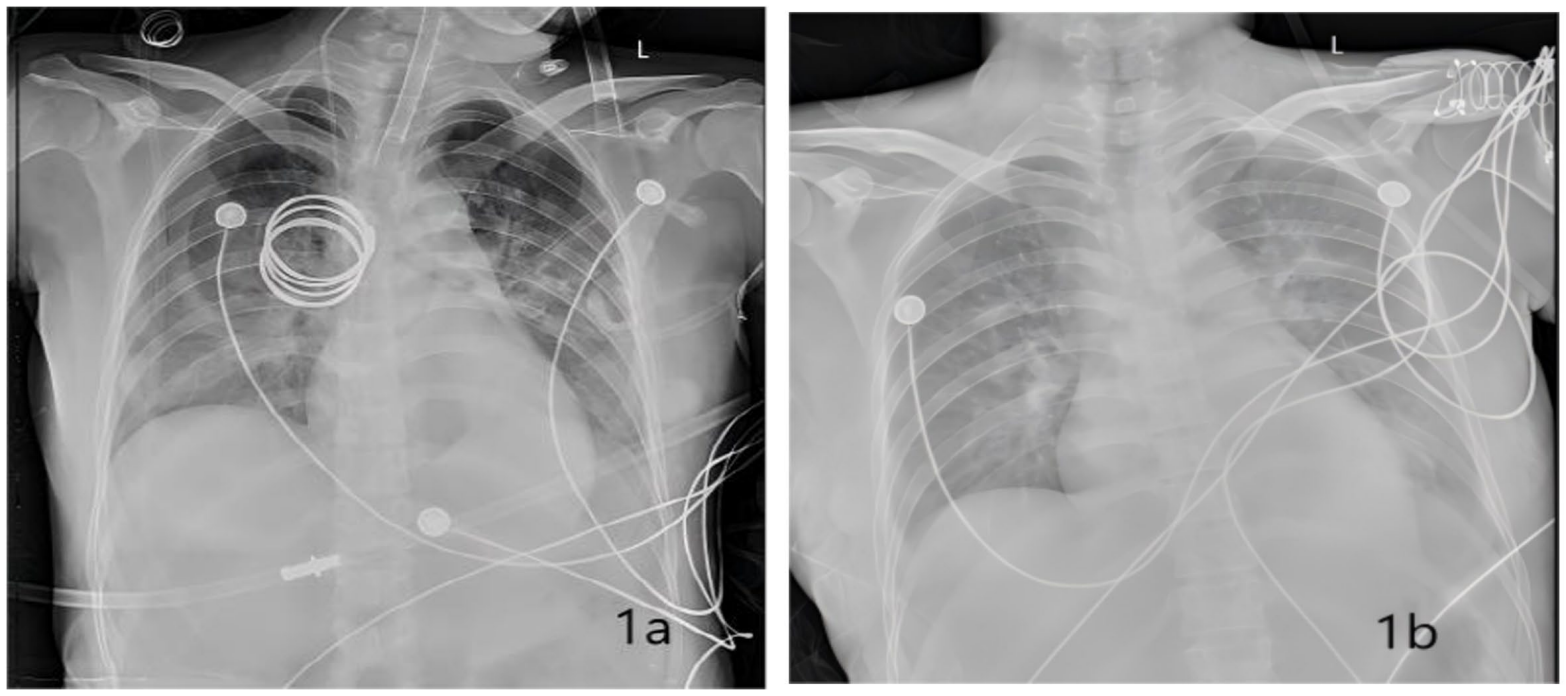
Bedside chest X-ray film findings of the patient. **(a)** Bilateral pulmonary exudation demonstrated on postpartum day 2. **(b)** Bilateral pulmonary exudation still demonstrated on postpartum day 5.

**Figure 2:**
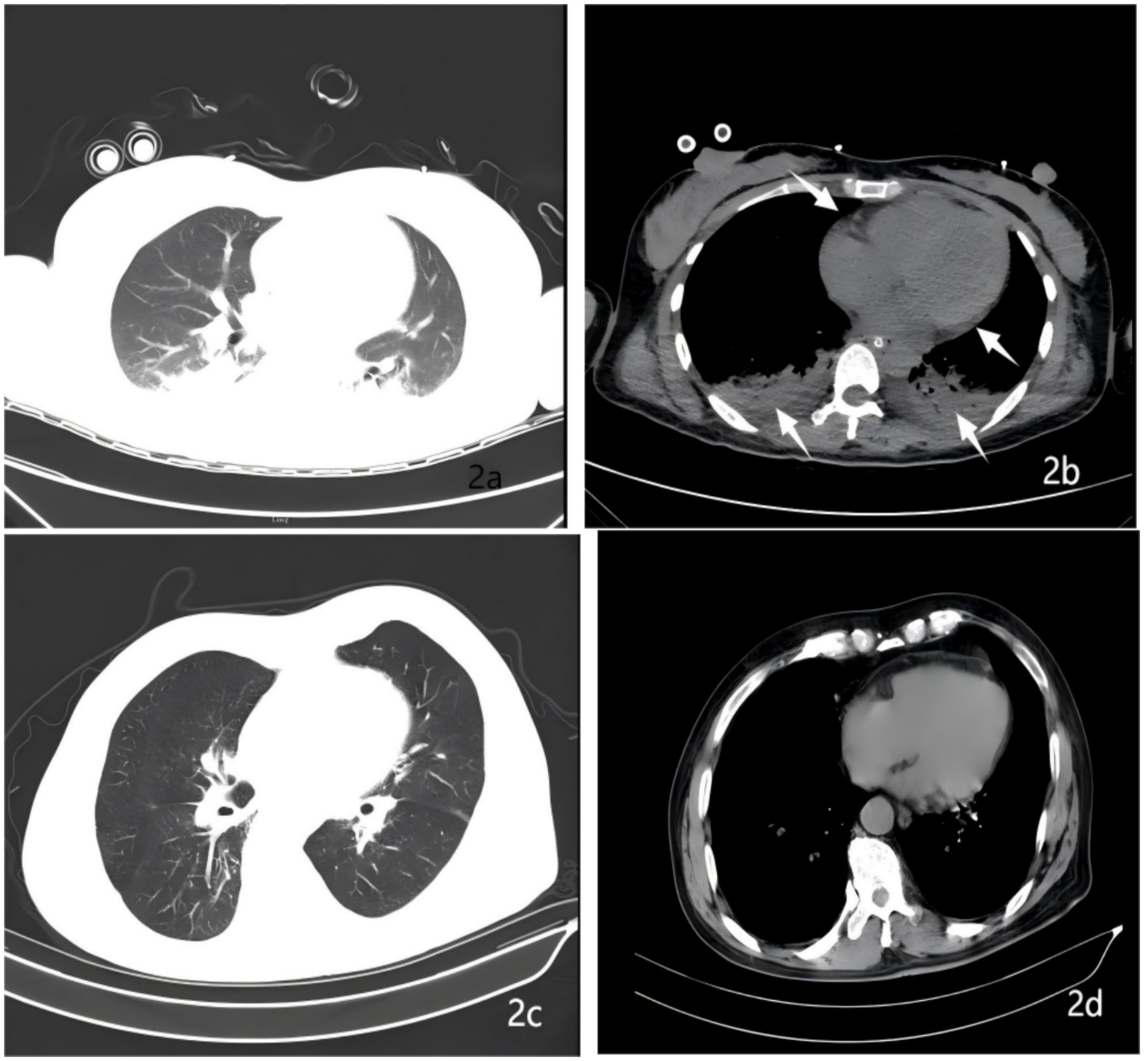
Pulmonary CT findings of the patient. **(a)** Bilateral pulmonary exudation on postpartum day 3. **(b)** Bilateral pleural effusion and pericardial effusion on postpartum day 3. **(c)** Reduced pulmonary exudation on postpartum day 7. **(d)** Improved pleural effusion and pericardial effusion on postpartum day 7.

**Figure 3:**
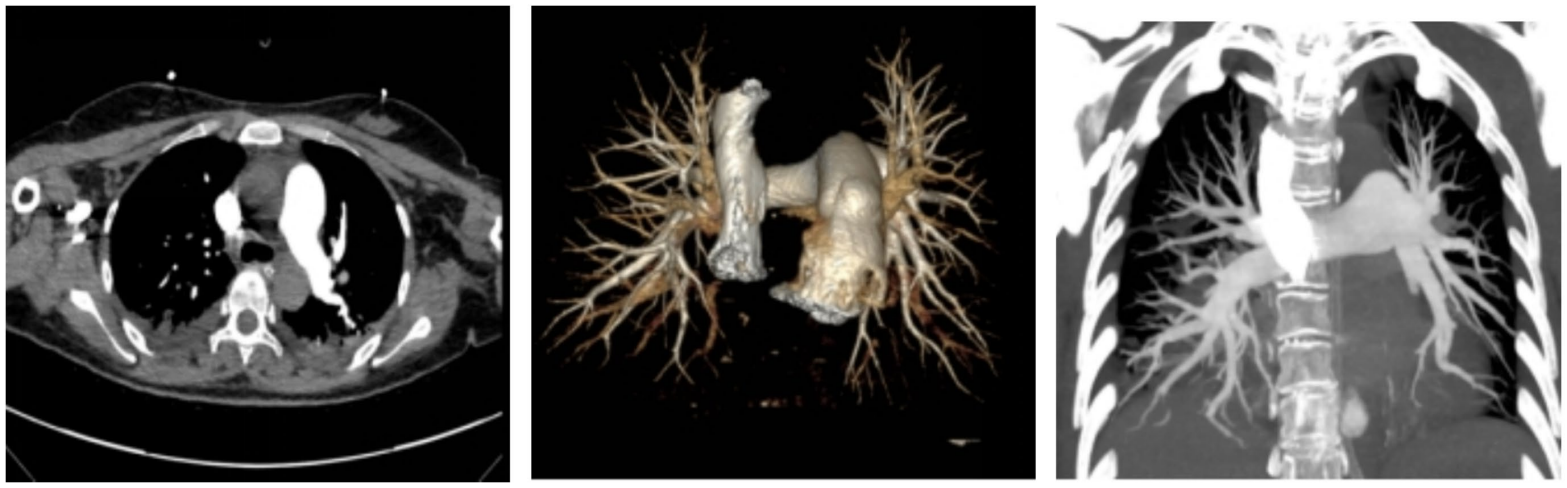
Pulmonary artery CTA findings of the patient on postpartum day 3. Demonstrating punctate filling defects in partial branches of the left pulmonary artery.

Hypoxemia caused by AFE was suspected. Endotracheal intubation and mechanical ventilation were performed immediately [A/C mode, respiratory rate 16 breaths/min, tidal volume 0.42 L/min, PEEP 5cmH₂O (1cmH₂O = 0.098 kPa), FiO₂ 100%] to maintain SpO₂ above 90%. On the 2nd postpartum day, a bedside chest X-ray showed bilateral pulmonary exudation (Figure 1a), suggesting pulmonary infection or pulmonary edema. Midazolam, remifentanil, and vecuronium were continued for analgesia, sedation, and muscle relaxation to ensure patient-ventilator synchrony; the fluid infusion rate was adjusted according to PICCO monitoring parameters and hourly urine output, and 20 mg. No chest CT was performed prior to the 3rd postpartum day, as the patient’s hemodynamic status was unstable in the acute phase; priority was therefore given to bedside resuscitation and invasive vital sign monitoring, and imaging examinations were deferred until her clinical condition had stabilized. On the 3rd postpartum day, a follow-up chest CT scan revealed bilateral pulmonary exudation (Figure 2a), bilateral pleural effusion, and pericardial effusion (Figure 2b); CTA (computed tomography angiography) showed embolism in some branches of the left pulmonary artery (Figure 3). On the 5th postpartum day, a follow-up bedside chest X-ray still indicated bilateral pulmonary exudation (Figure 1b). However, considering the patient’s current clear consciousness, stable respiratory and circulatory status, grade 4 muscle strength of the limbs, independent cough ability, and stable internal environment, she met the criteria for ventilator weaning; the patient had a positive spontaneous breathing trial (SBT), and ventilator weaning was successfully achieved with endotracheal tube extubation (14). High-flow oxygen therapy (FiO₂ 50%) was administered, and SpO₂ remained >95% (see Table 1). On the 7th postpartum day, a follow-up chest CT scan suggested a significant reduction in bilateral pulmonary exudation (Figure 2c), with marked improvement in bilateral pleural effusion and pericardial effusion (Figure 2d).

**Table 1:** Major organ function support measures and changes in key indicators of the patient.

Indicator	Before intervention	After intervention	Main support strategy
Oxygenation index	SpO2 85%, mechanical ventilation (FiO2 100%)	SpO₂ > 95%, high-flow nasal cannula (HFNC) (FiO₂ 50%)	Mechanical ventilation plus PEEP; image-guided combined with lung water monitoring for volume management and ventilator weaning
Myocardial contractility	35%	>50%	Inotropic support with milrinone plus dobutamine; nesiritide for cardiac function improvement; gradual tapering of vasoactive drugs
Coagulation function (PTA)	63.0%	102%	Early antifibrinolytic therapy plus component transfusion; timely initiation of enoxaparin anticoagulation after coagulation function recovery
Extravascular lung water index (EVLWI)	6 ml/kg	Returned to the normal range	Fluid restriction plus diuresis; refined volume management with albumin combined with crystalloid fluid

This table presents the baseline levels before intervention, improvement after intervention, and corresponding organ function support strategies of four core physiological indicators, namely oxygenation index, myocardial contractility, coagulation function (prothrombin activity, PTA), and extravascular lung water index (EVLWI), in the patient of this case. After intervention, the patient’s oxygenation status improved significantly, myocardial contractility recovered to the normal range, anticoagulant therapy was successfully initiated following the restoration of coagulation function, and EVLWI returned to the normal interval. The support strategies focused on respiratory support, inotropic therapy, coagulation regulation and refined volume management, and the gradual recovery of organ function was achieved through multimodal interventions.

### 3.2 Circulatory support therapy

After the onset of symptoms, the patient presented with shock. Fluid resuscitation was immediately performed with 500 mL dextran, and during endotracheal intubation, her heart rate dropped to 50 beats/min. Immediately, 2 mg epinephrine hydrochloride was administered intravenously by bolus injection, and norepinephrine at 2 μg/(kg·min) and metaraminol at 5 μg/(kg·min) were infused intravenously via a pump. After admission to the ICU, PICCO monitoring showed a cardiac index of 2.53 L/(min·m^2^), stroke volume index of 21 mL/m^2^, end-diastolic volume index of 518 mL/m^2^, stroke volume variation of 21%, systemic vascular resistance of 2,860 dyn·s·cm^−5^/m^2^, and extravascular lung water index of 6 mL/kg. Bedside echocardiography showed an inferior vena cava diameter of 0.8 cm and myocardial contractility of 35%, suggesting low cardiac output and hypovolemia. Fluid replacement with albumin plus normal saline was continued, and dobutamine at 8.2 μg/(kg·min) and milrinone at 0.31 μg/(kg·min) were added for intravenous pump infusion (15), while the dose of norepinephrine was reduced to 1 μg/(kg·min) and metaraminol infusion was suspended. The patient’s blood pressure became stable (Mean Arterial Pressure (MAP) > 65 mmHg), and tissue perfusion was improved compared with before (lactate: 5.55 → 2.23 mmol/L).

On the 4th postpartum day, recheck echocardiography suggested normal morphology and structure of all cardiac chambers, with mild mitral and tricuspid regurgitation, and myocardial contractility above 50%. Norepinephrine was gradually reduced and discontinued, and recombinant human brain natriuretic peptide (rhBNP) at 0.01 μg/(kg·min) was added for intravenous pump infusion (administered continuously for 3 days), along with continued combined inotropic therapy with dobutamine and milrinone (see Table 2). Meanwhile, the patient’s internal environment and urine output were monitored to ensure perfusion of vital organs and prevent pulmonary edema. This strategy highlighted the in-depth analysis of the mechanism of mixed shock caused by AFE and targeted intervention, realizing the model shift from pressure support to cardiac function reconstruction.

**Table 2:** Changes in cardiac function-related indicators and intervention summary during hospitalization and follow-up period.

Time point	Core cardiac function indicators	Targeted therapeutic interventions	Outcome
Day 1 (1 h post-delivery)	Myocardial contractility 35%; BP 66/33 mmHg; CI 2.53 L/(min·m^2^)	Epinephrine+norepinephrine+ metaraminol circulatory support; dobutamine+milrinone inotropic therapy; PICCO-guided volume resuscitation	Severe cardiogenic shock; intensive support initiated
Day 4	Myocardial contractility >50%; MAP≥65 mmHg; CI normalized	Taper metaraminol/norepinephrine; add rhBNP; maintain inotropic therapy	Cardiac function improved; shock corrected; tissue perfusion restored
Day 10	Myocardial contractility normal; BP stable (dobutamine only)	Discontinue norepinephrine; rhBNP + dobutamine support; optimized volume management	Stable cardiac function; shift to cardiac protection therapy
Day 15 (Discharge)	Myocardial contractility normal; BP 124/81 mmHg	Metoprolol, Sacubitril/Valsartan for long-term cardiac function improvement	Cardiac function basically recovered; met discharge criteria
10 Days Post-discharge	Myocardial contractility normal; BP stable	Oral cardiac protective drugs; moderate rehabilitation activity	Cardiac function improved; pulmonary embolism essentially absorbed
30 Days Post-discharge	Myocardial contractility normal; BP stable	Oral medication dose adjustment; regular cardiac function monitoring	Cardiac function fully recovered; ADL score 95; no sequelae

This table dynamically tracks the stepwise recovery of cardiac function from acute cardiogenic shock to full rehabilitation over 30 days of follow-up. It validates the efficacy of a physiological indicator-guided, stepped circulatory support strategy.

### 3.3 Management of coagulation disorders

After delivery, the obstetrics team administered 10 U oxytocin via intravenous infusion (16), 250 μg carboprost tromethamine via cervical injection, and 5 mg vitamin K1 via intramuscular injection to prevent postpartum hemorrhage (17). Based on the relevant laboratory results after symptom onset and combined with the clinical manifestation of vaginal bleeding, coagulopathy was clinically suspected.coagulation disorder was suspected. Therefore, 1 g TXA was added via intravenous infusion, and blood transfusion therapy (transfusion of packed red blood cells + fresh frozen plasma + cryoprecipitate) was administered (see Table 3).

**Table 3:** Changes in coagulation function-related indicators and intervention summary during hospitalization and follow-up period.

Time point	Core coagulation function indicators	Targeted therapeutic interventions	Outcome
Day 1 (1 h post-delivery)	PT 16.6 s (prolonged); PTA 63.0%; INR 1.33; PLT 109 × 10^9^/L	Tranexamic acid hemostasis; packed RBC + fresh frozen plasma + cryoprecipitate transfusion; hemorrhage prevention drugs	Acute AFE-related coagulation dysfunction; hemostatic therapy initiated
Day 3	PT/INR/PTA/Fib normal; PLT 109 × 10^9^/L	Enoxaparin 2000 U subcutaneously bid; MDT evaluation (HAS-BLED 2, Padua 7)	Coagulation function normalized; targeted anticoagulation initiated
Day 11	Coagulation indicators normal; PLT 248 × 10^9^/L	Continue enoxaparin; ambulation + ankle pump exercises to prevent thrombosis	Stable coagulation; lower extremity DVT resolved; no bleeding
Day 15 (Discharge)	All coagulation indicators normal; PLT 274 × 10^9^/L	Continue enoxaparin until 42 days postpartum; regular coagulation/D-dimer reexamination	Coagulation fully normalized; safe anticoagulation
10 Days Post-discharge	All coagulation indicators normal	Continue anticoagulation; dose adjustment based on reexamination	Coagulation-fibrinolysis balance restored; pulmonary embolism absorbed

This table illustrates the sequential management of coagulation function (hemostasis followed by anticoagulation) and the resolution of thrombotic complications. It confirms the safety and effectiveness of risk-stratified anticoagulation in AFE patients.

### 3.4 Management of complications

#### 3.4.1 Infection control

On the day of delivery, cefazolin was administered to the patient for infection prophylaxis. At 23:00, the patient’s maximum body temperature reached 39.1 °C. Combined with the patient’s inflammatory laboratory indicators and circulatory monitoring indicators (PICCO suggesting low cardiac output and hypovolemia), infection was suspected. Subsequent blood culture results were negative, which ruled out systemic bacteremia, yet pulmonary infection associated with AFE could not be completely excluded. The antibiotic was adjusted to meropenem 1.0 g administered intravenously every 8 hours (18). On the 3rd day, the patient’s body temperature returned to normal; on the 5th day, the patient’s respiratory and circulatory status was stable, and inflammatory indicators improved compared with before. Meropenem was discontinued, and cefoperazone sodium and sulbactam sodium 4.5 g administered every 8 h was used instead; on the 7th day, chest CT suggested improvement in pulmonary exudation and bilateral pleural effusion compared with before; on the 10th postpartum day, the patient’s condition was stable, and she was transferred to the obstetrics ward for further treatment.

#### 3.4.2 Deep venous thrombosis (DVT)

The patient’s DVT risk score upon ICU admission was 13 points, classifying her as a high-risk population for DVT. Due to the patient’s coagulation disorder, hemostatic therapy and blood transfusion were administered, making her unsuitable for pharmacologic DVT prophylaxis. Intermittent pneumatic compression therapy was given for 18 h per day; on the 4th day, vascular ultrasound indicated bilateral calf muscular venous thrombosis, and intermittent pneumatic compression therapy was suspended; on the 3rd day, the patient’s rechecked indicators showed significant improvement: platelet count 109 × 10^9^/L, PT 13.2 s, PTA 102%. Enoxaparin 2000 U administered subcutaneously every 12 h was given for anticoagulant therapy (19); on the 11th day, rechecked vascular ultrasound showed no thrombosis in the bilateral calf muscular veins.

**Figure 4:**
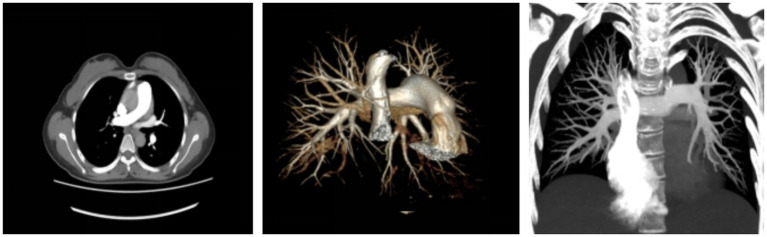
Pulmonary artery CTA findings of the patient on postpartum day 24. Demonstrating well-visualized bilateral pulmonary artery branches, with emboli in partial branches of the left pulmonary artery essentially absorbed.

After active rescue treatment, the patient’s circulation was stable on the 4th postpartum day (only rhBNP and dobutamine were administered for inotropic support); she successfully weaned off the ventilator and had the endotracheal tube removed on the 5th day, and was stably transferred out of the ICU on the 10th day; she was discharged with improved condition on the 15th day. At the follow-up visit 10 days after discharge, pulmonary artery CTA showed good opacification of bilateral pulmonary artery branches, no obvious filling defects, and basically absorbed embolism in some branches of the left pulmonary artery (Figure 4). At the 30-day follow-up after discharge, the patient was conscious, with an activity of daily living (ADL) score of 95, no discomfort such as dyspnea or chest tightness, and normal mental and cognitive function.

## 4 Discussion

AFE is a rare and often fatal obstetric complication. Relevant studies report an incidence of 0.99 cases per 100,000 deliveries for AFE, with a mean diagnostic latency of 85.51 min for fatal AFE cases (20). The patient in this case achieved a favorable treatment outcome. Reflecting on the management process, we believe that on the basis of conventional multidisciplinary collaboration, this case achieved significant improvements in treatment efficiency and outcomes through systematic optimization of early identification pathways, implementation of a dynamic coagulation-anticoagulation balance strategy, and image-guided precise intervention—providing new insights and approaches for AFE clinical practice.

AFE is characterized by abrupt onset, life-threatening progression, and unpredictability, which can lead to severe adverse outcomes such as maternal and fetal disability or even death. Thus, saving the lives of the mother and fetus depends on the rapid identification of this syndrome (21). Currently, there is no internationally unified diagnostic criteria or validated laboratory diagnostic basis for AFE. Classic diagnostic indicators for AFE include high-risk factors, nonspecific prodromal symptoms, respiratory and circulatory failure, coagulopathy, multiple organ failure, and findings from transthoracic echocardiography or transesophageal echocardiography (1). The patient presented with sudden dyspnea and cyanosis, accompanied by decreased oxygen saturation, shock, and vaginal bleeding immediately after delivery. Combined with laboratory findings, the patient was considered to have acute hypoxemia, hypotension, and coagulopathy—meeting the clinical diagnostic criteria for AFE.

The Wells scale was used for preliminary screening of pulmonary embolism at the initial diagnosis, with a score of 6 points (high probability), and the pregnancy-adapted YEARS criteria was further applied to exclude alternative diagnoses. This combined diagnostic approach provided a quick and standardized basis for the early suspicion of AFE complicated by pulmonary embolism and effectively avoided the delay of diagnosis caused by nonspecific clinical symptoms.

The management of AFE requires close collaboration of the MDT to improve treatment efficiency (22). The patient had no typical manifestations in the early examination. The MDT quickly ruled out other fatal acute conditions based on experience. Through dynamic monitoring of laboratory indicators and combination with bedside ultrasound, the final diagnosis of AFE was confirmed. The patient received treatments such as life support, symptomatic treatment, and organ function protection (22).

After delivery in the obstetrics department, the patient suddenly developed severe hypoxemia, respiratory failure, and circulatory failure. Given the constellation of acute clinical manifestations, immediate multidisciplinary emergency management was initiated. Due to the rapid progression of AFE, collaborative rescue by multiple departments and disciplines was required. Therefore, we immediately initiated MDT emergency treatment, completing endotracheal intubation, mechanical ventilation, fluid resuscitation, and blood pressure elevation at the bedside. Based on the initiation of MDT, this case broke through the traditional “departmental consultation” model and established a “structured response unit” centered on critical obstetrics, covering specialized positions in hemodynamics, coagulation management, and imaging evaluation. Relying on real-time monitoring technologies such as PICCO, bedside ultrasound, and rapid coagulation testing, closed-loop management from diagnostic hypothesis →treatment implementation → effect evaluation was completed at the emergency bedside, significantly reducing decision-execution delay and reflecting systematic innovation from “institutional collaboration” to “technological integration.”

During the management of this case, our hospital utilized a critical obstetric care center linked by the “5-minute emergency life chain.” Centered on obstetrics, this center integrates collaboration from multiple disciplines, including critical care medicine, anesthesiology, transfusion medicine, interventional radiology, cardiology, ultrasonography, vascular surgery, hematology, radiology, and medical administration to provide urgent and efficient treatment for critically ill parturients. When the patient developed critical signs such as decreased SpO₂ and hypotension, obstetricians promptly identified the possibility of AFE in the parturient, quickly coordinated with anesthesiology and critical care medicine departments for bedside emergency treatment, and then transferred the patient to the ICU. The critical care medicine team further contacted relevant departments to conduct MDT management, formulated the treatment plan, performed comprehensive management of the patient’s respiratory, circulatory, coagulation, and renal systems, corrected acid–base imbalance, prevented infection, and avoided multiple organ failure (23). A “response-prediction-control” tripartite model was implemented in this case, upgrading the approach from passive rescue execution to proactive risk prediction and process control. This model advanced the “consultation-based collaboration” of traditional MDT to a closed-loop management of “integrated decision-making with real-time feed back,” significantly improving the precision and efficiency of treatment.

The patient developed acute hypoxemia, manifested as sudden dyspnea, cyanosis of the lips, and decreased SpO₂. Amniotic fluid entering the maternal bloodstream triggers an anaphylactoid immune response, activates the systemic inflammatory response, increases vascular endothelial permeability, and induces pulmonary edema; concurrently, pulmonary vasospasm and microthrombi markedly reduce pulmonary blood flow, leading to severe ventilation/perfusion (V/Q) mismatch and subsequent acute hypoxic respiratory failure (24). A comprehensive strategy of “respiratory support plus inflammatory response control” was adopted to break the pathological vicious cycle and improve the patient’s prognosis (25). Upon the onset of hypoxemia, emergency bedside endotracheal intubation and mechanical ventilation were initiated immediately; dexamethasone combined with epinephrine was administered intravenously to inhibit mast cell degranulation and cytokine release, thereby attenuating the systemic inflammatory response; invasive blood pressure monitoring and PICCO were used to guide fluid management, preventing and correcting pulmonary edema; pulmonary infection was prevented through standardized antibiotic use, ventilator-associated pneumonia (VAP) prevention bundles, early respiratory rehabilitation, and postural drainage. The patient successfully underwent ventilator weaning and endotracheal extubation on the 5th postpartum day.

The main causes of circulatory failure in AFE patients include distributive hypovolemic shock due to systemic inflammatory response, obstructive shock due to pulmonary hypertension, and hypovolemic shock due to extensive hemorrhage caused by DIC; additionally, endotoxins and inflammatory cytokines directly suppress myocardial contractility, leading to cardiac dysfunction (23). Therefore, treatment targeted three core issues in AFE-related circulatory failure: vasodilation, myocardial suppression, and visceral ischemia. In the early phase, a combined intervention strategy of “vasopressor-inotrope-organ protection” was implemented using epinephrine, norepinephrine, and dopamine to maintain a mean arterial pressure (MAP) ≥ 95 mmHg. Simultaneously, volume resuscitation was performed with rapid crystalloid infusion followed by colloid supplementation: central venous pressure (CVP), cardiac output, extravascular lung water index, and global end-diastolic volume were monitored to prevent and alleviate pulmonary edema.

The patient developed sudden vaginal bleeding post-delivery. Although the bleeding volume was within the normal postpartum range, the concurrent presence of shock and hypoxemia was consistent with organ injury secondary to acute systemic coagulopathy. Meanwhile, laboratory tests (PT, INR, D-dimer, 3P test, TAT, and PIC) indicated concurrent coagulation factor consumption and hyperfibrinolysis, reflecting an acute coagulation-fibrinolysis imbalance; although the platelet count was normal, the aforementioned laboratory findings suggested ongoing platelet consumption; combined with the presence of AFE as an etiological trigger, the patient met the diagnostic criteria for acute hemorrhagic coagulopathy (26).

Given that the underlying mechanism was AFE-induced DIC, treatment focused on etiological intervention combined with correction of coagulation abnormalities (anticoagulation and coagulation factor replacement). In the early phase, when bleeding risk was predominant, hemostatic therapy with tranexamic acid and component transfusion (platelets, fresh frozen plasma, and cryoprecipitate) was administered, with close monitoring of coagulation function (27). On the 3rd postpartum day, re-evaluation showed improved coagulation function, with vaginal bleeding reduced to approximately 200 mL.

Pulmonary embolism is a core pathological event driving the rapid deterioration of AFE patients, and early identification and intervention of pulmonary embolism are crucial for improving prognosis. Pulmonary CTA on the 3rd postpartum day indicated the presence of pulmonary embolism. Given the sudden onset of dyspnea and shock in the early postpartum period, combined with the patient’s medical history, we prioritized AFE-related pulmonary embolism over isolated thrombotic pulmonary embolism in the differential diagnosis (28). Although acute massive hemorrhage and DIC can temporarily reduce the risk of thromboembolism, the postpartum period is inherently characterized by a physiological hypercoagulable state (29). The pulmonary embolism observed on day 3 in this case was more likely associated with AFE-related coagulation disorders, particularly during the transition phase from DIC-induced hypocoagulability to hypercoagulability, rather than being caused by TXA. Evidence from large-scale randomized controlled trials and meta-analyses has confirmed that the standard short-course administration of tranexamic acid does not increase the risk of thrombosis (30, 31). Notably, the timing of pulmonary embolism occurrence in this patient was fully consistent with the well-recognized high-risk period for thrombotic complications secondary to AFE-related coagulation disorders. During the acute phase of AFE, the primary risk is bleeding due to coagulation factor consumption by DIC. Therefore, routine anticoagulation is not recommended for pulmonary artery obstruction; instead, treatment focuses on oxygenation, shock correction, and coagulation factor replacement (plasma and cryoprecipitate).

On the 3rd postpartum day, screening for DVT revealed calf muscular venous thrombosis. With a Padua risk score of 7, the patient was classified as high-risk for secondary thrombotic pulmonary embolism. The initiation of pharmacologic anticoagulation for AFE patients with concurrent thrombosis represents a critical clinical decision that requires reconciliation with established clinical guidelines. The American College of Obstetricians and Gynecologists (ACOG) recommends withholding systemic anticoagulation during the acute phase of AFE (32), as acute consumptive coagulopathy and life-threatening bleeding are the primary clinical concerns. However, current thrombosis management guidelines (33) mandate a formal bleeding risk assessment for patients with a history of coagulopathy, with pharmacologic anticoagulation permissible once DIC is resolved and bleeding risk is low—this formed the core rationale for the treatment plan adopted in this case. The clinician followed a structured, risk-stratified anticoagulation protocol (33) with the decision to initiate low-molecular-weight heparin (LMWH) strongly supported by three key clinical factors: the complete resolution of DIC (normalized coagulation parameters), a low HAS-BLED score of 2 (34) (indicating minimal bleeding risk), and a high Padua score of 7 (signaling a high risk of recurrent thromboembolism). This intervention model, based on serial parameter monitoring and dual assessment of bleeding-thrombosis risk, overcomes the traditional contraindication of anticoagulation in AFE and enables the safe implementation of individualized anticoagulation therapy.

For severe AFE patients with massive pulmonary embolism who are unresponsive to conventional supportive treatment, percutaneous catheter embolectomy and extracorporeal membrane oxygenation (ECMO) are important salvage treatment options in clinical practice (35, 36). Percutaneous catheter embolectomy is mainly suitable for patients with hemodynamic instability caused by proximal pulmonary artery embolism and no obvious contraindications to interventional therapy (35), which can quickly remove pulmonary artery emboli, relieve pulmonary vascular obstruction, and improve cardiac output and oxygenation status. ECMO plays a critical supportive role in AFE complicated by severe respiratory and circulatory failure, which can effectively maintain the patient’s oxygenation and hemodynamic stability, create a valuable window for the treatment of the primary disease, and reduce the mortality of severe AFE patients (36). ECMO is especially indicated for AFE patients with refractory hypoxemia and cardiogenic shock that cannot be corrected by mechanical ventilation and high-dose vasoactive drugs.

In this case, the emboli were limited to partial branches of the left pulmonary artery without massive proximal embolism, and the patient’s hemodynamic and oxygenation status were quickly corrected and stabilized by conventional respiratory support, circulatory intervention and coagulation factor replacement. Thus, the patient did not meet the clinical indications for percutaneous catheter embolectomy or ECMO application, which also confirms that timely and standardized conventional supportive treatment is the core of improving the prognosis of non-massive AFE complicated by pulmonary embolism. In addition, the integrated use of imaging technology, including pulmonary CTA and lower extremity venous ultrasound throughout the treatment process, also reflects a paradigm shift from empirical therapy to visualized and targeted treatment, which is highly consistent with the precision medicine direction of modern obstetric critical care.

## 5 Conclusion

AFE progresses rapidly; once suspected, emergency treatment for AFE should be initiated immediately. Multidisciplinary rescue management is recommended. This article reports a case of a parturient who developed sudden AFE after forceps-assisted delivery, complicated by secondary pulmonary embolism and multiple organ dysfunction. Through the rapid initiation of emergency procedures by the multidisciplinary team, combined with respiratory and circulatory support, correction of coagulation disorders, infection control, and targeted anticoagulation therapy, the patient’s condition was ultimately reversed.

This case confirms the core AFE management principles of “early identification-multidisciplinary collaboration-individualized intervention,” and particularly emphasizes that AFE should be prioritized in the differential diagnosis when sudden cardiopulmonary failure combined with DIC occurs during high-risk procedures such as forceps-assisted delivery. Pulmonary artery CTA plays a key role in identifying pulmonary embolism lesions and guiding the timing of anticoagulation, while dynamic monitoring of coagulation indicators and organ function provides important basis for adjusting treatment strategies. The successful management of this case further indicates that a standardized emergency system and multidisciplinary collaboration are decisive factors for improving the prognosis of AFE.

## Data Availability

The original contributions presented in the study are included in the article/supplementary material, further inquiries can be directed to the corresponding author/s.
